# Personalized symptom management: a quality improvement collaborative for implementation of patient reported outcomes (PROs) in ‘real-world’ oncology multisite practices

**DOI:** 10.1186/s41687-020-00212-x

**Published:** 2020-06-17

**Authors:** Doris Howell, Zeev Rosberger, Carole Mayer, Rosanna Faria, Marc Hamel, Anne Snider, Denise Bryant Lukosius, Nicole Montgomery, Mindaugas Mozuraitis, Madeline Li, the iPEHOC Collaborative Team, Katherine George, Zahra Ismail, Adriana Krasteva, Ashley Kushneryk, Lorraine Martelli, Alyssa Macedo, Julia Park, Lesley Moody, Lisa Barbera, Pat Giddings, Subhash Bhandari, Linda Tracey, Julie Szasz

**Affiliations:** 1 University Health Network (Princess Margaret Cancer Centre), 610 University Health Network Room 15-617, Toronto, ON M5G 2M9 Canada; 2 University of Toronto, Toronto, ON Canada; 3 Lady Davis Institute for Medical Research, Jewish General Hospital, Montreal, Quebec Canada; 4 McGill University, Montreal, Quebec Canada; 5 Health Sciences North Research Institute, Sudbury, ON Canada; 6 Montreal West Island Integrated University Health & Social Services Center, St. Mary’s Hospital, Montreal, Quebec Canada; 7 Psychosocial Oncology Department, McGill University Health Centre, Montreal, Quebec Canada; 8 Juravinski Cancer Centre, Hamilton, ON Canada; 9 McMaster University, Hamilton, ON Canada; 10 Cancer Care Ontario, Toronto, ON Canada; 11 Center for Addiction and Mental Health, Toronto, ON Canada

**Keywords:** Cancer, Patient reported outcomes, Health care utilization, Real world implementation, QI collaborative, Oncology practices, Multisite

## Abstract

**Background:**

Little research has focused on implementation of electronic Patient Reported Outcomes (e-PROs) for meaningful use in patient management in ‘real-world’ oncology practices. Our quality improvement collaborative used multi-faceted implementation strategies including audit and feedback, disease-site champions and practice coaching, core training of clinicians in a person-centered clinical method for use of e-PROs in shared treatment planning and patient activation, ongoing educational outreach and shared collaborative learnings to facilitate integration of e-PROs data in multi-sites in Ontario and Quebec, Canada for personalized management of generic and targeted symptoms of pain, fatigue, and emotional distress (depression, anxiety).

**Patients and methods:**

We used a mixed-methods (qualitative and quantitative data) program evaluation design to assess process/implementation outcomes including e-PROs completion rates, acceptability/use from the perspective of patients/clinicians, and patient experience (surveys, qualitative focus groups). We secondarily explored impact on symptom severity, patient activation and healthcare utilization (Ontario sites only) comparing a pre/post population cohort not exposed/exposed to our implementation intervention using Mann Whitney U tests. We hypothesized that the iPEHOC intervention would result in a reduction in symptom severity, healthcare utilization, and higher patient activation. We also identified key implementation strategies that sites perceived as most valuable to uptake and any barriers.

**Results:**

Over 6000 patients completed e-PROs, with sites reaching 51%–95% population completion rates depending on initial readiness. e-PROs were acceptable to patients for communicating symptoms (76%) and by clinicians for treatment planning (80%). Patient experience was better than the provincial average. Compared to the pre-population, we observed a significant reduction in levels of anxiety (*p* = 0.008), higher levels of patient activation (*p* = 0.045), and reduced hospitalization rates (12.3% not exposed vs 10.1% exposed, *p* = 0.034). A pre/post population trend towards significance for reduced emergency department visit rates (14.8% not exposed vs 12.8% exposed, *p* = 0.081) was also noted.

**Conclusion:**

This large-scale pragmatic quality improvement project demonstrates the impact of implementation strategies and a collaborative improvement approach on acceptability of using PROs in clinical practice and their potential for reducing anxiety and healthcare utilization; and improving patient experience and patient activation when implemented in ‘real-world’ multi-site oncology practices.

## Background

Personalized medicine is changing the landscape of cancer care [1]. Cherny et al. propose that personalized medicine should encompass biologically personalized therapeutics, as well as “individually tailored whole-person care that is at the bedrock of what people want and need when they are ill” [2]. Patient reported outcomes (PROs) are an important aspect of personalized medicine [3] that can enable person-centered ‘whole’ person care and improve health outcomes when they are used by clinicians [4, 5]. Indeed, systematic reviews of randomized clinical trials have shown that PROs improve patient/provider communication and may improve other health outcomes such as quality of life and reduced emergency department visits [6–10]. A survival advantage has also been shown in randomized controlled trials (RCTs) for electronic PROs (e-PROs) when clinicians are prompted to address adverse events between clinic visits via alerting systems [11]. If we are to realize the benefit of PROs on health outcomes on a larger scale, we need to move beyond RCTs and drive optimal uptake of PROs data for clinically meaningful use in healthcare decisions and for person-centered patient management [12–14]. Unfortunately, little evidence has been generated with regards to implementation of PROs in ‘real-world’ settings and it is unclear what implementation strategies work best to facilitate uptake in practice [15, 16].

Despite a decade of experience of deploying e-PROs in 14 Regional Cancer Centers (RCCs) in Ontario, Canada for distress screening [17, 18] using the Edmonton Symptom Assessment System revised version (ESAS-r) [19], the use of this data in patient management is sub-optimal [20, 21]. This is not surprising as PROs implementation in ‘real-world’ cancer care is complex, requiring reconfiguration of clinic workflow, changes in both clinicians’ practice behaviors and multidisciplinary team collaboration to address PRO scores [22]. Use of strategies to overcome the multiple implementation barriers (e.g. lack of perceived value, difficulty in interpreting PRO data, poor integration in clinical workflow) that can impede a quality response to PRO data is required [23–25]. Thus, it is recommended that best practices in knowledge translation and implementation science methods be used to promote uptake and integration of PRO data in clinical practices [26, 27].

We initiated a Quality Improvement Collaborative, the Improving Patient Experience and Health Outcome Collaborative (iPEHOC), to drive uptake of e-PRO data by clinicians for person-centered management of symptoms in multi-site oncology practices in Ontario and Montreal, Quebec. Although the evidence for Quality Improvement Collaborative approaches has been equivocal, there is strong face validity that they are valuable for improving targeted clinical processes and a range of health outcomes such as symptom severity [28]. We are unaware of other studies that have used this approach for PROs implementation in multi-site practices. Our aims were to: 1) Evaluate uptake of e-PROs measured as percent of completed e-PROs from baseline to project end as run charts, acceptability/use from the perspective of patients/clinician, and changes in patient experience of care; 2) Explore impact on symptom severity, patient activation, and emergency department visit (ED) and hospitalization (H) rates (Ontario only). We hypothesized that the iPEHOC intervention would reduce symptom severity, healthcare utilization, and be associated with higher levels of patient activation; and 3) Identify implementation strategies considered by sites as essential for successful uptake of e-PROs in clinical practice.

## Methods

We used a mixed-methods (quantitative surveys and qualitative data) program evaluation design to evaluate change in care processes. Qualitative focus groups of patients and clinicians in each site were conducted post-intervention to obtain their perspective of the e-PROs and their use in clinical care (reported in a separate paper). To explore impact on health and system outcomes we compared a pre-implementation population cohort (non-exposed to iPEHOC) to a post-implementation population cohort (iPEHOC exposed). Participating regional cancer centres and disease site clinics in Ontario included: 1) Princess Margaret Cancer Centre (PM), a comprehensive RCC in an urban setting, in lung and sarcoma disease site clinics; 2) Northeast Cancer Center (NECC), serving rural and remote regions, in the chemotherapy, radiotherapy, supportive care and palliative care clinics, and 3) Juravinski Cancer Centre (JCC), a midsized RCC serving urban and rural populations, in central nervous system and gynecology clinics. RCCs in Montreal, Quebec included: 1) Saint Mary’s Hospital Centre (SMHC), a small community hospital, in medical oncology clinics; 2) Segal Cancer Center, a comprehensive regional cancer centre at the Jewish General Hospital (JGH) in gynecologic clinics; and 3) McGill University Health Centre (MUHC), a large academic RCC, in lung clinics. Ethics approval for a multi-site study was obtained from the University Health Network Research Ethic Board (REB) (REB #14–8525-CE) followed by approvals in all regional cancer centres in both provinces.

### iPEHOC implementation intervention

**Fig. 1:**
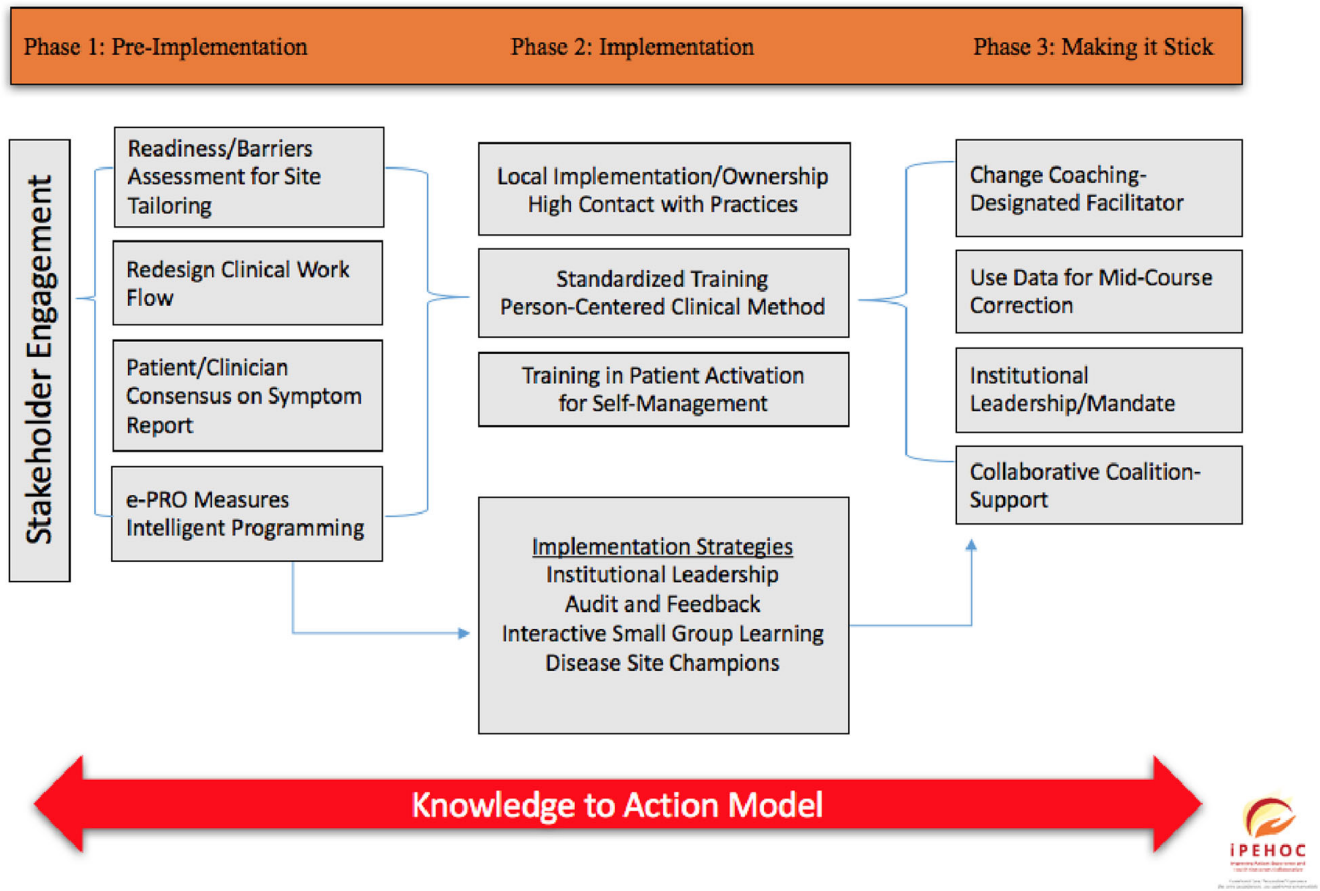
iPEHOC Phases of Implementation and Key Strategies

Implementation is defined as the use of specific activities and strategies that promote the adoption and integration of evidence-based interventions and change practice [29]. We used a three-phased, implementation approach (Fig. 1, Table 1) guided by integrated knowledge translation [30], the Knowledge-to-Action framework [31] and principles of a collaborative QI approach [28]. Integrated knowledge translation is defined as an ongoing relationship between researchers and decision-makers to foster uptake of innovations in practice [30].

**Table 1:** Implementation Phases and Implementation Strategies

Phases	Implementation and Change Management Strategies
Phase 1: Setting the Stage	a. Promote awareness of the need for change through presentations-create a compelling vision for PROs on patient outcomes (i.e. rounding) b. Engage key stakeholders at each site (patients, clinicians, IT leaders, administrative and disease site leaders) in a local implementation team (coalition) to facilitate practice change and integrate PROs in workflow c. Complete readiness assessment to characterize current support for PROs implementation and barriers to tailor implementation strategies d. Interactive educational meetings and focus groups to reach consensus on a visual format for the PROs symptom report (summary of scores). e. Designation of disease site champions and opinion leaders to facilitate practice change within each site (internal practice change facilitation).
Phase 2: Active Implementation	a. Standardized clinician training using simulated case-based scenarios with standardized patients to model integration of PROs for personalized communication and treatment planning, tailoring of interventions to symptom scores, and activation of patients (how to change). b. Interactive case-based education in disease site clinics to discuss response to scores and tailoring of guideline recommendations to practice (how to perform effectively). One-to-one role modeling if needed. d. Educational brochures/videos targeted to increasing patient knowledge of PRO use for monitoring and guiding symptom self-management. e. Foster integration of patient symptom management guidelines in patient education sessions as part of standardized approach.
Phase 3: Making it Stick	a. Engaged administrative leaders and provincial quality leads in sites/provinces to champion the change and performance accountability. b. Disease site champions/opinion leaders acted as change management facilitators at monthly disease site team business meetings. c. Collaborative all sites meetings with centralized program manager to share implementation strategies (external practice change facilitation). d. Use of audit and feedback as an implementation strategy to show progress in screening rates and change in symptom scores. e. Early discussion of sustainability and plans for sustaining the change.

### Phase 1 (3 months: pre-Implementation/setting the stage)

#### Technical considerations

We built on an existing ESAS-r electronic platform and added four psychometrically valid and reliable, pan-Canadian endorsed e-PRO measures for multidimensional assessment of targeted symptoms of pain (Brief Pain Inventory-BPI) [32], fatigue (Cancer Fatigue Scale-CFS) [33], depression (Prime Health Questionaire-PHQ-9) [34], and anxiety (Generalized Anxiety Disorder-GAD-7) [35] (Additional Attachment 1, iPEHOC measurement system). Internal logic was built into the platform to trigger the multidimensional e-PROs based on previously established ESAS-r cut-scores of > 3 ESAS-r anxiety to trigger GAD-7; > 2 ESAS-r depression to trigger PHQ-9 [36], > 4 ESAS-r pain to trigger BPI, and > 4 ESAS-r fatigue to trigger the CFS [37]. We also built time-frame logic into the system for triggering these e-PROs at 21 days for anxiety and depression, and at 7 days for pain and fatigue, based on consensus amongst clinicians of the appropriate time-frame to observe a change from a treatment plan. A single item Quality of Life scale [38] was also included, and at Princess Margaret and Quebec sites, the Social Difficulties Inventory-21 (SDI-21) [39].

e-PROs were collected on stationary kiosks or tablets, with clinic receptionists and/or volunteers prompting patients to complete upon clinic registration. e-PRO data was scored in real-time and fed-back to clinicians (and patients) as a printed summary report (a graph of scores over time was also accessible in electronic medical records) of severity scores for nine ESAS-r plus targeted iPEHOC symptoms for use in the clinical encounter in person-centered communication (Additional File 2, iPEHOC symptom report). An initial galvanizing meeting was held with Collaborative members (provincial quality cancer agency leads and decision-makers for Ontario and Quebec, clinicians, patients, disease site leads) followed by meetings with disease site teams and patient partners in each site to catalyze a compelling vision for the change (i.e. key evidence of benefits). At the initial collaborative meeting sites worked with disease site teams to develop an implementation blueprint that incorporated recommended implementation strategies to facilitate uptake of PROs in routine clinical care. Implementation teams were formed in each site to: 1) facilitate practice change using champions and case-based, educational outreach sessions, 2) devise a change plan tailored to site enablers and barriers identified at baseline through team completion of an adapted version of the Organizational Readiness Survey (ORS) [40], 3) map current workflow and reconfigure clinical processes, i.e. workflow/team collaboration, to integrate e-PRO data in clinic encounters, 4) share learnings in monthly collaborative meetings to spread successful implementation strategies based on social learning and diffusion of innovation theories [41].

### Phase 2 (*6 Months,* active *Implementation)*

In this phase, disease-site champions (identified by disease site leads as early adopters ESAS-r, used in practice, and respected by peers) worked alongside project coordinators and site implementation teams to facilitate practice change for use of e-PRO data in patient management. Champions help to facilitate and catalyze change through persuasive communication and interpersonal skills [42, 43]. During this phase, we used evidence-informed, multifaceted implementation strategies [44, 45] inclusive of core training of all clinicians (target of minimum of 70%), monthly case-based educational outreach sessions, audit and feedback reports, and tracked progress using monthly run charts to show rates of e-PROs completion in each RCC.

Core training included sessions on 1) interpretation of e-PRO scores and benefits of use and 2) case-based video-based simulations using clinicians paired with standardized patients that modelled a person-centered clinical method [46] for embedding of e-PRO data in the clinical encounter. The videos demonstrate use of e-PRO data scores for opening the dialogue with patients, developing a shared agenda and treatment plan based on problems that were prioritized as “mattering most” to patients and important to be addressed by clinicians in this clinic visit, use of e-PRO data to guide intervention selection and manage problems based on best practices in pan-Canadian evidence-based practice guidelines [47–49], and advising patient actions for symptom self-management. Additionally, to foster patient activation, in partnership with the Canadian Partnership Against Cancer we developed and disseminated videos to patients about how to interpret e-PRO scores and use of e-PRO reports in communication with clinicians [50] and distributed patient facing symptom management guidelines for use in site-based patient education [51].

Audit and feedback reports were tailored to each site but with common data elements including e-PRO completion rates, % of patients who met ESAS-r cut-offs and were required to complete the multidimensional e-PROs, and symptom change scores for discussion at monthly disease site meetings (Additional File 3: Audit and feedback reports). Audit and feedback data stimulates change in clinician behaviour’s through peer pressure [52]. Typically, audit and feedback data is reported back to individual clinicians with comparison to peers, but participating sites desired an overall disease site performance report. iPEHOC sites tracked the number of educational sessions delivered and staff attendance/discipline and use of implementation strategies (educational outreach, audit and feedback, etc.) on excel spreadsheets monthly for monitoring of implementation fidelity.

### Phase 3 (3 months-making it stick*: Embedding and Sustaining Use in Practice)*

In this phase, case-based, interactive educational outreach sessions were ongoing to further facilitate embedding of e-PRO data use in clinical practice; and sustain the practice change. Interactive educational sessions work by sustaining momentum, changing health professionals’ awareness and beliefs about current practice and perceived subjective norms and builds their self-efficacy (confidence) and skills [53].

### Process and exploration of impact on outcomes

#### Aim 1

The rate of e-PROs completion (number of completed e-PROs in participating clinics/number of patients eligible to complete) were tracked from baseline to project end as monthly run charts (rate of completion/population who could have completed). Descriptive statistics were used to summarize acceptability/use of e-PROs via surveys of patients (Patient Acceptability Survey-PAS) and clinicians (Clinician Satisfaction Survey-CSS). Surveys were developed specifically for iPEHOC based on items in other surveys [54]. Patient experience was assessed by completion of two-items from the Ambulatory Oncology Patient Satisfaction Survey (AOPPS) [55], to assess satisfaction with care received for managing emotional concerns and physical symptoms. The PAS was distributed in iPEHOC participating clinics waiting rooms over a 14-day period at 4 months (mid-point) and post-implementation. A target sample of a minimum of 50 completed surveys/site was pre-determined based on sampling for QI purposes [56]. Clinicians were invited to complete the CSS at midpoint and end of implementation via an email sent from the site lead with an embedded link to the survey with a 7-day e-mail reminder sent based on a modified Dillman survey methodology [57].

#### Aim 2

To explore impact on symptom severity, intra-individual change scores using ESAS-r data for a 6-months pre-implementation population cohort (non-iPEHOC exposed) were compared to scores for an ESAS-r plus IPEHOC (exposed) population cohort in the 6-months during the final months of implementation (Ontario sites only). Symptom scores were rank ordered based on their occurrence in time and a symptom change slope of outcome on time using linear regression were generated to account for systematic person-specific deviations such as serial correlation, time-varying medical events, and irregular measurement times. The mean slopes of the change scores were subjected to unequal samples ANOVA with the RCC site and the observation window as categorical variables. Using a similar timeframe, a Mann Whitney U-test was used to evaluate change in levels of patient activation using the brief Patient Activation Measure (PAM) [58]. The PAM measures knowledge, skills, and confidence for self-management and segments patients into one of four progressively higher levels of patient activation as follows: Level 1 (lack knowledge/confidence for managing health), Level 2 (knowledgeable, unsure about actions to take), Level 3 (knowledgeable, initiating health self-management skills), and Level 4 (using health behaviours, but struggle under stress).

In Ontario sites only, we compared the % of the population in the baseline observation window cohort (90 days pre-iPEHOC/non-exposed) to the % of the population in the 90 days post-iPEHOC implementation observation window cohort (exposed) admitted to the ED or hospitalized (H) within 30 days of an e-PRO report in that timeframe. Data sources for health utilization outcomes included the Symptom Management Reporting Database (SMRD) [59], which captures e-PRO data for Ontario RCCs, Canadian Institute for Health Information (CIHI): National Ambulatory Care Reporting System (NACRS) [60], Discharge Abstracts Database (DAD) [61] and the Activity Level Reporting (ALR) database of CCO [62]. NACRS records all visits to the ED and hospitalization, whereas the Activity Level Reporting database captures all visits to RCPs in Ontario for visit identification.

## Results

### Completion rates

**Fig. 2:**
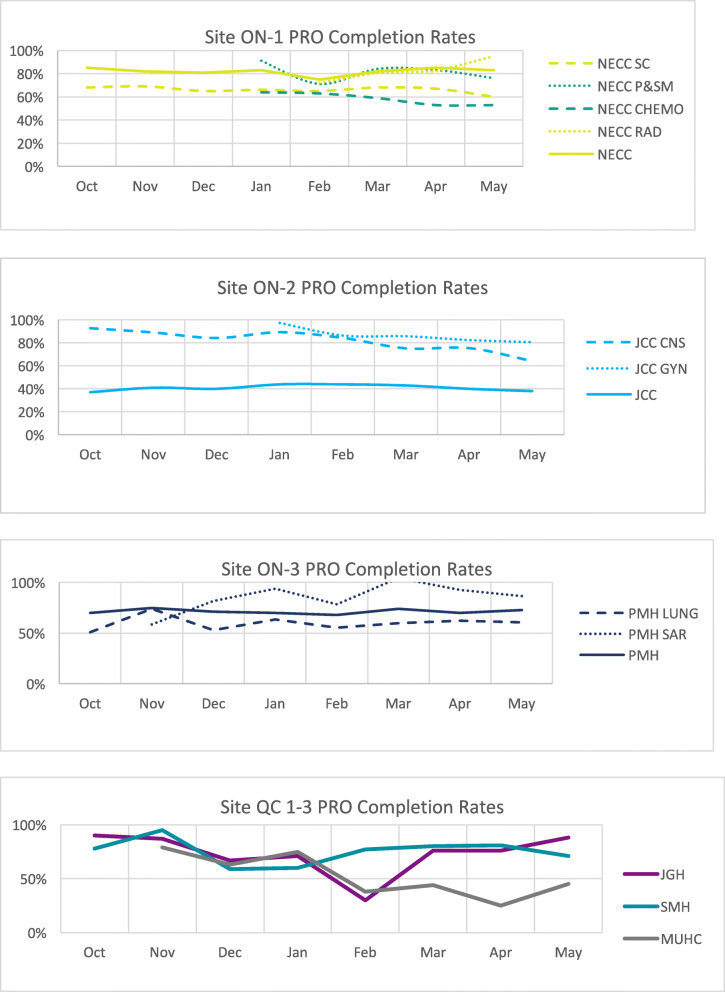
PRO Completion Rates-Baseline to Project End

Sites had varying baseline rates of using any e-PROs prior to iPEHOC implementation. In Ontario sites, NECC and JCC had baseline ESAS-r completion rates of 75% and 37% respectively, and PM had baseline completion rates of 86%. We observed an increase in e-PRO completion rates over time across the six sites; or rates were maintained if initially high at project start (Fig. 2). Time to complete ESAS-r plus all four e-PROs took on average 9 min, 56 s (tracked electronically in the platform). Overall, 6000 e-PROs were completed across sites.

### Acceptability/use and patient experience

Results from the PAS (Ontario sites, *n* = 182, Montreal sites *n* = 54) indicated that 67% Ontario and 79% of Montreal patients respectively rated the e-PROs as acceptable for enabling communication about symptoms with their health care team. Compared to average population rates in Ontario for the two-items from AOPPS there was a shift in patient experience from pre/post implementation (Table 2). Of the 62 clinicians (50% nurses, 26% physicians, 36% allied health) who completed the CSS, slightly more than half (58%) felt the e-PROs had value and were used for symptom management in clinic visits and most (75–85%) were very satisfied with their ability to respond (data not shown). Slightly more than a third (36%) thought e-PROs prolonged clinic visit times. However, only 25% of respondents from NECC speciality clinics reported e-PROs had value as clinicians felt the e-PROs were redundant to comprehensive assessments already performed.

**Table 2:**
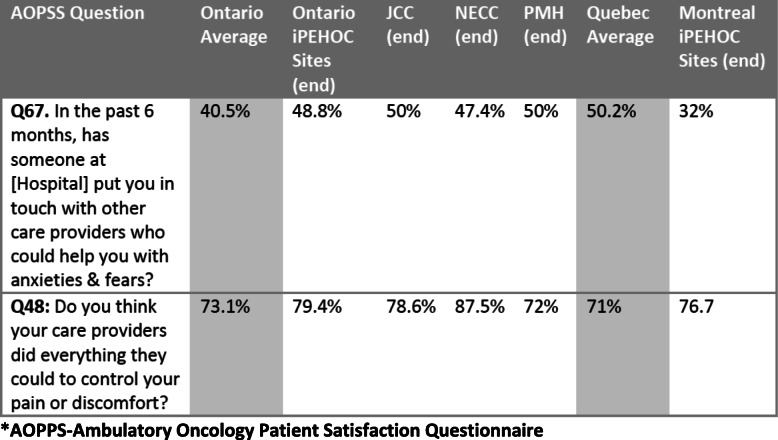
Patient Experience Compared to Provincial Standards-2 Items.

*AOPPS-Ambulatory Oncology Patient Satisfaction Questionnaire

### Symptom severity

**Fig. 3:**
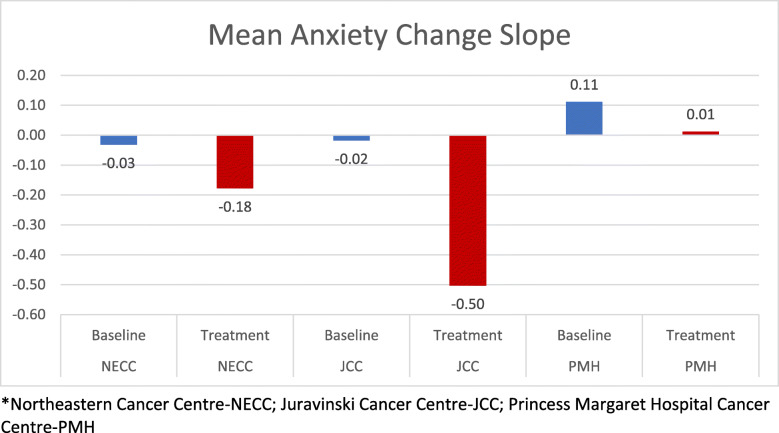
Slope of Change in Mean Anxiety

We examined slopes of intra-individual change scores for all targeted e-PRO symptoms in Ontario sites only (fatigue, pain, depression, anxiety), but only significant slopes for change in anxiety were observed. A significantly larger reduction in anxiety was observed in the iPEHOC exposed population, compared to the pre-iPEHOC non-exposed cohort, *p* = 0.004 (Fig. 3). This finding was not as marked at PM, since GAD-7 was already in use pre-implementation, whereas the marked reduction in the anxiety distress slope in NECC and JCC may be indicative of the value added from the ESAS-r plus iPEHOC e-PROs in these sites.

### Patient activation

**Fig. 4:**
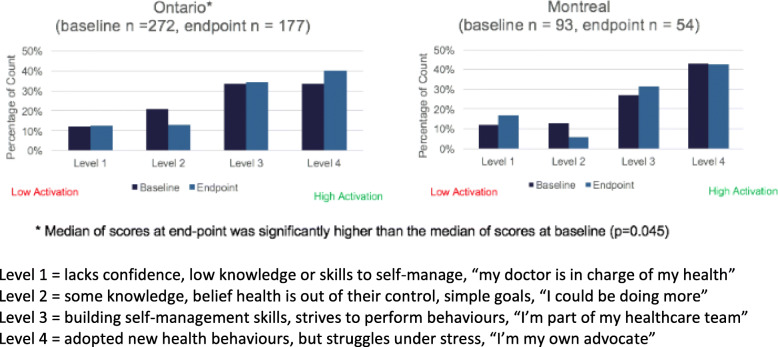
Pre/Post Patient Activation Scores for Disease Sites Combined for Ontario and Montreal

A shift to higher levels of activation and a small but statistically significant increase in median scores on the Patient Activation Measure from baseline to end-point was observed (*p* = 0.045) in Ontario sites combined but not in Montreal sites (Fig. 4). This finding may be due to the increased exposure to ESAS-r in Ontario since 2007 and the small sample size in Montreal.

### Health care utilization

For all Ontario sites combined, a small significant reduction was shown in hospitalization rates (*p* = 0.034) in the pre-implementation population (30 days after completion of the e-PRO report) (*n* = 299, 12.3%) compared to a post-population rate of 10.1% (exposed, *n* = 162). A trend towards significance was also observed for emergency department visit rates (*p* = 0.081) in the pre-population (*n* = 359, 14.8%) compared to the post-population rate of 12.8% (*n* = 205) (Table 3). The greatest contribution to the overall emergency department visit rates came from disease site clinics targeted in the Juravinski Cancer Centre, where ED visits were reduced from 20.1% to 12.7% (*p* = 0.051), and for hospitalizations in their disease site populations in the Juravinski Cancer Centre (11.8% to 4.9%, *p* = 0.014) and in the lung and sarcoma cancer population in the Princess Margaret Cancer Centre (14.8% to 10.6%, *p* = 0.041).

**Table 3:**
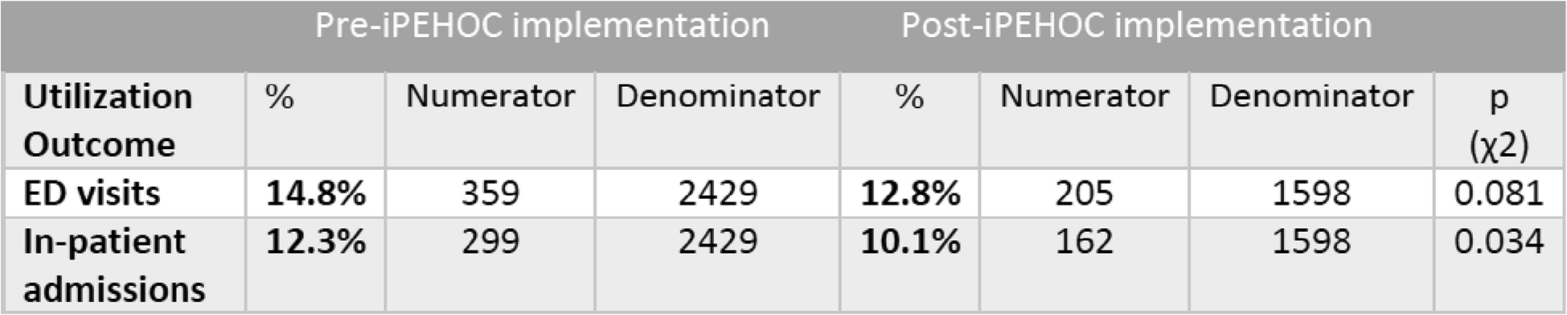
Pre/Post Emergency Department Visits and Hospitalization Rates (Ontario only).

### Implementation strategies

Implementation strategies identified by sites as key for facilitating uptake of e-PROs are shown in Table 4. A supportive leadership structure that establishes PROs use as performance metric, building clinician capacity and confidence in interpreting and responding to PRO data using case-based education and educational outreach, adaptive technology to trigger multidimensional e-PROs when screened positive based on ESAS-r and output reports that are easy to interpret were identified as key factors for successful uptake. Also, broad engagement of all stakeholders, high contact with practices, ongoing monitoring and use of audit and feedback, respected peers as champions, and site coordinators skilled in knowledge translation and facilitating practice change were considered key to successful implementation. Sites also identified the collaborative approach as helpful for sharing of ideas and gaining support in dealing with resistance to practice change. Our iPEHOC implementation methods toolkit is available online and recommendations were integrated for use in the Ontario provincial e-PROs framework to guide implementation steps in other PROs work [63]. A checklist was developed as part of the iPEHOC toolkit for use in guiding implementation in other organizations (Additional File 4: iPEHOC implementation checklist).

**Table 4:** Key Implementation Strategies for PROs Uptake in Practice

	Strategy	Approach
Training and Coaching	**Induction Process Clinicians & Patients**	• Train clinicians in PRO score interpretation, how to integrate in clinical encounter for communication about “what matters most to patient” and use in patient management and devising shared treatment plans (standardized patient modeling in video simulations and in site visits) • Educate patients on how to use PROs in physical symptom and emotional distress self-management and use PROs report for communication with clinicians in clinic visit • Model a person-centred approach for use by clinicians in treatment plans and for engaging patients in taking actions for symptom self-management
	**Feedback of Interpretable Reports**	• PROs easy to complete/not burdensome to patients (< 10 min to complete) • Complete prior to clinic visit at 1st point of contact to ensure summed report available at clinic visit • Summarized scores in easy to interpret format (patient and clinician) i.e. red flag severe scores with integration in electronic patient record and/or printed for access/use in clinic visit
	**Educational Outreach/Coaching**	• Ongoing case-based educational outreach sessions to facilitate use of best practice interventions for managing PRO identified problems; protocols for best practices aligned to scores & clear pathways for referral • Champions respected by peers to encourage uptake in practice; model use in practice/peer learning • Clinician/patient orientation includes standardized training on PROs and ‘how’ to use in communication and for self-management • Integrate PROs in patient self-management guides and patient education pamphlets • Project managers skilled in facilitating practice change
Leadership Support & Accountability	**Infrastructure and Technology**	• Electronic completion available in different languages-data infrastructure at local site • Data infrastructure and designated IT support for timely management of technology problems; tablets accessible to patients at first point-of-contact i.e. blood labs; configure PROs for seamless completion if initial positive screen • Adaptive technology to trigger e-PROs for multidimensional measures if met cut-offs on ESAS-r • Usability field test prior to full implementation
	**Engagement of Key Stakeholders**	• All stakeholders (clinicians, patients/families, leadership) involved in selection of relevant PROs; and input into “look and feel” of output reports • System mapping by leadership to exploit critical pathways in patient care, resource requirements
	**Governance and Accountability**	• Performance accountability for use of PROs in patient management-monitored in QI programs • Alignment of objectives and strategic goals of the organization and daily rapid-cycle improvement priorities • Leadership sets PROs use as a priority performance metric in clinical care (rates of completion)
	**Resources**	• Patients have sufficient support to facilitate PROM completion (volunteers, technical assistance) • PROs coordinator with knowledge translation and change management skills for practice uptake
Disease Site Ownership	**Reconfigure Workflow**	• Reconfigure work flow to ensure integration and access to PRO reports at clinical encounter • Patient flow for completion of PROs and normalized as part of clinical care and patient pathways
	**Audit and Feedback**	• Performance reports designed with stakeholders and feedback to disease site teams for population based QI • Audit and feedback reports emphasize change in scores as a proxy for appropriate intervention • Systems to track progress and identify targets for improvement
	**Team Working**	• Team “working” planned and reconfigured to address “what score level” must be addressed and by whom i.e. nurse counselling of patient management of fatigue • Ongoing work in disease site teams to drive optimal use of PROs in care and patient management-institutionalizing the change

## Discussion

Globally, greater attention has been focused on the use of e-PROs in health care organizations to achieve person-centered and tailored supportive care [64]. Embedding of PROs for guiding healthcare decision-making and patient management requires use of implementation strategies to facilitate practice change and redesign of care processes and workflow if improved health outcomes are to be acheived [65]. The American Society of Clinical Oncology (ASCO) has recommended routine use of e-PROs as a health policy priority for oncology practices [66], yet little evidence beyond passive dissemination of e-PRO information systems has been generated as to how to embed this data for use in ‘everyday’ oncology practices. A recent review identified only 3 reports of ‘real-world’ implementation of e-PROs in clinical practice and none of these studies used knowledge translation or implementation strategies to facilitate integration of PROS for personalized patient management [15]. Our study makes a novel contribution to the literature by identifying a collaborative approach and person-centred clinical training method for embedding of e-PRO data in the clinical encounter for patient management and for patient activation in symptom management. Additionally, we have identified key implementation strategies that promote successful uptake and applied these in diverse disease sites and urban, regional and remote cancer settings. Like most other studies, we found that e-PROs were acceptable to patients as it gives them a ‘voice’ to communicate their experience of the impact of cancer and treatment [67]. A shift in patient experience regarding emotional concerns and symptoms may be indicative of changes in care processes and uptake of the e-PROs in patient care. Clinical trial data show that e-PROs when used in clinical care improves quality of life, time on chemotherapy, reduces health care use and may improve survival if monitored and responded to between visits, but there is still a need for real-world evidence of impact [68].

Despite the limitations of small sample sizes, heterogeneity, and possible within site clustering in pre/post population cohort comparisons, we demonstrated the potential impact of multi-faceted implementation strategies on reducing anxiety and health care utilization but future large-scale trials are needed. A reduction in anxiety shown for iPEHOC may suggest patients felt more confident their symptoms would be addressed by clinicians using e-PRO data. This effect was not found for other targeted symptoms of pain, depression, fatigue, which likely require more targeted interventions [69]. The positive change in emergency department visit rates and hospitalization found for use of ESAS-r alone [70] suggests that early management of symptom and emotional distress may mitigate escalation [71].

While we noted a shift towards higher levels of patient activation in pre/post population comparisons, we used passive dissemination of information about PROs to patients and emphasized participatory communication approaches to promote patient activation in clinician training, but there is a need for greater attention to use of PROs for activating patients in symptom self-management; and as an essential component of PROs implementation [72].

Implementation problems are described as messy, complex and wicked [73]. Our experience certainly echo’s this sentiment as we found that facilitating implementation across multiple disease site teams was challenging since disease site teams function as their own microsystem within the larger Regional Cancer Centres (meso-program level system) and provincial cancer system (macro cancer system), each of which have their own unique local barriers to uptake of e-PROs. Additionally, our measurement of outcomes was impacted by the ‘noise’ of implementation and ‘real-world’ problems such as simultaneous health system restructuring in Montreal, Quebec. Not surprisingly, the complexity of implementing PROs for use in the 'everyday' practice of clinicians in cancer settings has been previously described as “easier said than done” in other demonstration projects [74].

## Conclusion

Successful implementation of e-PROs can transform health care towards achieving better health outcomes [75], but this requires use of knowledge translation and implementation science methods for integration in work flow and embedding in the ‘everyday’ practice of clinicians for personalized patient management. Future large scale pragmatic trials to assess effectiveness, long term sustainability and cost-effectiveness of PRO use in patient management are needed. Implementation of e-PROs for patient management may be facilitated if identified as a performance metric [76] and for payment for performance in value-based care [77].

## Supplementary information

**Additional file 1.**

**Additional file 2.**

**Additional file 3.**

**Additional file 4.**

## Data Availability

The datasets generated and/or analyzed during the current study are not publicly available as they are owned by the respective provincial authority and are only accessible through this entity; and restrictions may apply.

## Abbreviations

AOPPS: Ambulatory oncology patient satisfaction survey
ALR: Activity level reporting
ASCO: American society of clinical oncology
BPI: Brief pain inventory
CFS: Cancer fatigue scale
CSS: Clinician satisfaction survey
DAD: Discharge abstracts database
ED: Emergency department
ESAS-r: Edmonton symptom assessment system-revised
GAD-7: Generalized anxiety disorder questionnaire
iPEHOC: Improving patient experience and health outcomes collaborative
iKT: Integrated knowledge translation
H: Hospitalized
JCC: Juravinski Cancer centre
JGH: Jewish general hospital (JGH)
KTA: Knowledge to action framework
QI: Quality improvement
MUHC: McGill University health centre
NACRS: National ambulatory care reporting system
NECC: Northeastern Cancer Centre
ORS: Organizational readiness survey
PAS: Patient acceptability survey
PAM: Patient activation measure
PHQ-9: Prime health questionnaire
PM: Princess margaret cancer centre
PROs: Patient reported outcomes and electronic PROs (e-PROs)
RCT: Randomized controlled trial
RCC: Regional cancer centre
SDL: Social Difficulties inventory
SMHC: St. Mary’s healthcare centre
SMRD: Symptom management reporting database
