# Supplementary figures and images for: Personalized symptom management: a quality improvement collaborative for implementation of patient reported outcomes (PROs) in ‘real-world’ oncology multisite practices
Source: J Patient Rep Outcomes. 2020 Jun 17;4:47. doi: 10.1186/s41687-020-00212-x (PMC7300168; doi:10.1186/s41687-020-00212-x)

**Additional File 1: iPEHOC Electronic Patient Reported Outcome Measurement System**

**
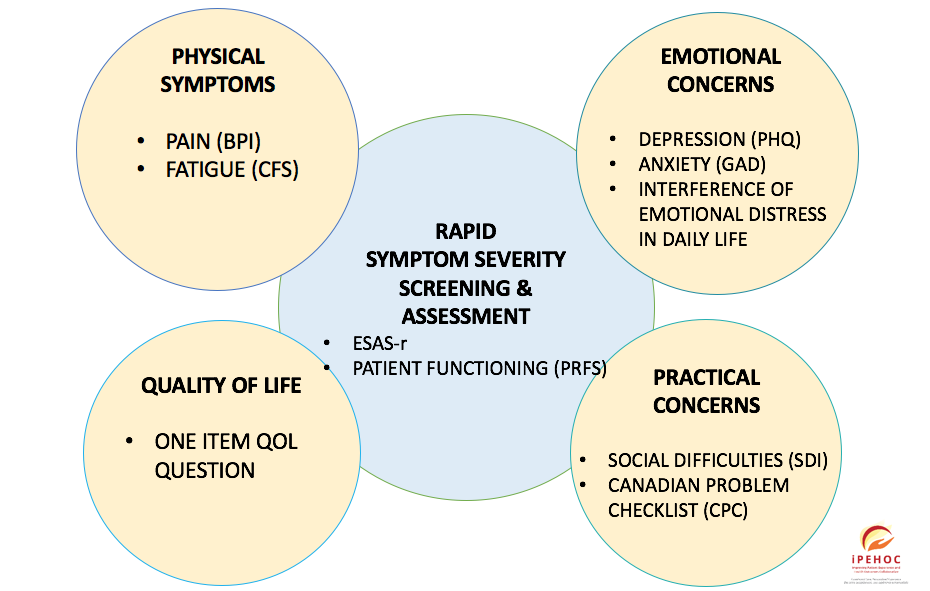
**

Supplement: Supplementary file 1 — Additional file 1. [file 41687_2020_212_MOESM1_ESM.docx]

**Additional File 2: iPEHOC Symptom Report**


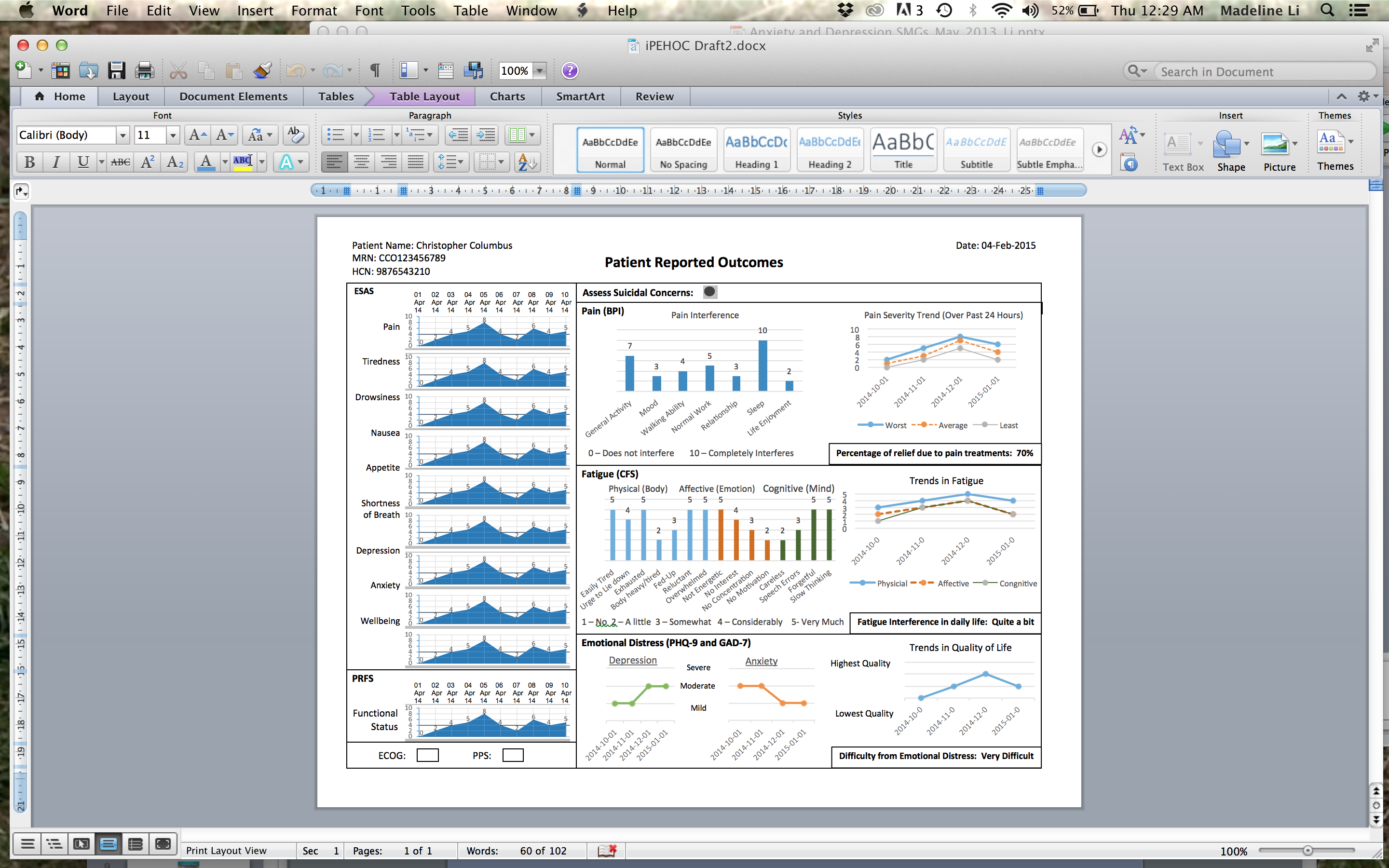

Supplement: Supplementary file 2 — Additional file 2. [file 41687_2020_212_MOESM2_ESM.docx]

**Additional File 3: iPEHOC Intervention Sample Audit and Feedback Report**


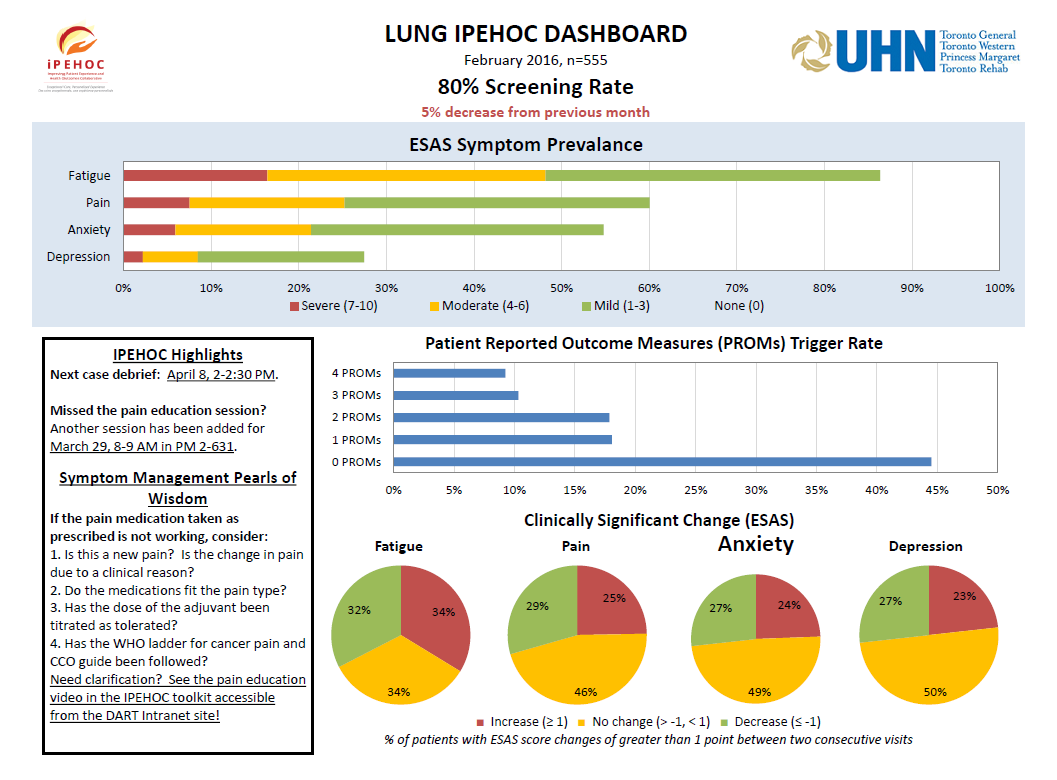

Supplement: Supplementary file 3 — Additional file 3. [file 41687_2020_212_MOESM3_ESM.docx]
