# Supplementary material for: Personalized symptom management: a quality improvement collaborative for implementation of patient reported outcomes (PROs) in ‘real-world’ oncology multisite practices
Source: J Patient Rep Outcomes. 2020 Jun 17;4:47. doi: 10.1186/s41687-020-00212-x (PMC7300168; doi:10.1186/s41687-020-00212-x)
Supplement: Supplementary file 4 — Additional file 4. [file 41687_2020_212_MOESM4_ESM.docx]

Additional File 4: Improving Patient Experience and Health Outcomes Collaborative (iPEHOC)

Implementation Checklist

**Preparing for the Change:**

- Define the change required at the site and modify any project-wide plans (e.g. communication, education), introductory communication materials, and checklists to the local context.
- Obtain sponsorship from appropriate administrative and executive leadership.
- Administrative or executive lead introduce the change to staff who will be involved in change management via modified communications.
- Invite key members to sit on site Implementation Team and establish a regular meeting schedule.
- Determine Implementation Team and collaborative governance and terms of reference.
- With input from Implementation Team and stakeholders involved in change, conduct an assessment of the current state of the site, including:
  - Stakeholder readiness, sponsorship of the change, and potential resistance/barriers to change;
  - Current clinic flow, processes and use of patient-reported outcomes;
  - Assessment and referral processes and resources;
  - Existing education and patient support resources that can be leveraged;
  - Risks associated with the change and potential mitigation strategies.
- Map clinic workflow and processes and reconfigure for PROMs integration in workflow.
- Raise awareness of the upcoming change in site staff, leveraging existing education opportunities (e.g. rounds, team meetings) and posting or distributing communication materials for buy-in.
- Clarify and educate staff members on responsibilities and accountabilities for new processes (e.g. registration, flow of PROMs output for use in clinics).
- Establish regular team meetings to review implementation progress and foster practice change.
- Develop evaluation plan for assessing outcomes on an ongoing basis and providing feedback to site stakeholders to stimulate mid-course corrections.

**Managing the Change:**

- During the change, allow for shared staff learning and coaching through regular team meetings (e.g. huddles, case review meetings).
- Maintain a system for regular updates from implementation team members (e.g. regular huddles, case review meetings), ensuring that feedback is reported directly to site leads and via most efficient channels to administrative leads, based on established governance structure.
- Address feedback where possible through adjustments to process or implementation approach.
- Report feedback regularly to project leadership at Steering Committee Meetings, making recommendations about project-wide changes.
- Follow a clearly specified implementation blueprint and change plan to guide the change that incorporates evidence-based implementation strategies and how these are operationalized.

**Reinforcing the Change:**

- Assess and evaluate data related to PROMs, PREMs, staff measures, and acute care and emergency room use (where available); use data to inform decision-making and to adjust implementation strategies.
- Use established meeting structures and staff educational resources (e.g. site meetings, rounds) to provide feedback on project progress.
- Develop a sustainability plan
